# Genome-wide RNAi screen reveals the E3 SUMO-protein ligase gene *SIZ1* as a novel determinant of furfural tolerance in *Saccharomyces cerevisiae*

**DOI:** 10.1186/1754-6834-7-78

**Published:** 2014-05-23

**Authors:** Han Xiao, Huimin Zhao

**Affiliations:** 1 Department of Chemical and Biomolecular Engineering, University of Illinois at Urbana-Champaign, Urbana, IL 61801, USA; 2 Departments of Chemistry, Biochemistry, and Bioengineering, Institute for Genomic Biology, University of Illinois at Urbana-Champaign, Urbana, IL 61801, USA

**Keywords:** Furfural tolerance, RAGE, *Saccharomyces cerevisiae*, *SIZ1*, SUMO E3 ligase

## Abstract

**Background:**

Furfural is a major growth inhibitor in lignocellulosic hydrolysates and improving furfural tolerance of microorganisms is critical for rapid and efficient fermentation of lignocellulosic biomass. In this study, we used the RNAi-Assisted Genome Evolution (RAGE) method to select for furfural resistant mutants of *Saccharomyces cerevisiae*, and identified a new determinant of furfural tolerance.

**Results:**

By using a genome-wide RNAi (RNA-interference) screen in *S. cerevisiae* for genes involved in furfural tolerance, we identified *SIZ1*, a gene encoding an E3 SUMO-protein ligase. Disruption of *SIZ1* gene function by knockdown or deletion conferred significantly higher furfural tolerance compared to other previously reported metabolic engineering strategies in *S. cerevisiae.* This improved furfural tolerance of *siz1Δ* cells is accompanied by rapid furfural reduction to furfuryl alcohol and leads to higher ethanol productivity in the presence of furfural. In addition, the *siz1Δ* mutant also exhibited tolerance towards oxidative stress, suggesting that oxidative stress tolerance related proteins may be under the SUMO regulation of SIZ1p and responsible for furfural tolerance.

**Conclusions:**

Using a genome-wide approach, we identified a novel determinant for furfural tolerance, providing valuable insights into the design of recombinant microbes for efficient lignocellulose fermentation.

## Background

There is a growing interest worldwide in using lignocellulose, the most abundant renewable biomass, to replace cereal substrates in the production of biofuels and biochemicals [1,2]. However, efficient fermentation of lignocellulosic hydrolysates is limited by inhibitors that are inevitably released during pretreatment and hydrolysis of lignocellulosic substrates [3]. Formed by dehydration of pentoses during dilute acid pretreatment of lignocelluloses, furfural is one of the major inhibitors present in lignocellulosic hydrolysates [4]. The toxicity of hydrolysates correlates with furfural concentration, with 1 to 5 g/L of furfural leading to complete growth inhibition of *Escherichia coli*, *Zymomonas mobilis* and *Saccharomyces cerevisiae*, significantly reducing the yield and productivity of desired products [5-8]. Although physical or chemical strategies for furfural detoxification can be adopted during fermentation, the additional equipment and time required increase the production costs [9]. Thus, improving furfural tolerance in microorganisms would provide a cost-effective means for lignocellulose fermentation.

*S. cerevisiae* is the most widely studied model organism for furfural tolerance and has higher furfural tolerance compared to other potential biofuel and biochemical production hosts [10-12]. Furfural modulates expression of genes involved in a variety of general stress responses in *S. cerevisiae*, including oxidative stress, nutrient starvation, DNA damage, unfolded protein response, as well as osmotic and salt stress [11]. However, whether and how these genes contribute to furfural tolerance is unknown [13]. To date, the known mechanism of furfural detoxification is its reduction into the less toxic furfuryl alcohol through reduced nicotinamide adenine dinucleotide phosphate(NADPH)-dependent enzymes [13,14]. Attributed to the significant increase in mRNA abundance and protein expression level observed in adapted *S. cerevisiae* under stress challenge, the NADPH-dependent oxidoreductases ADH7p and YKL071Wp were found to be responsible for furfural detoxification [15,16]. Due to limited knowledge on the mechanisms of furfural toxicity towards cells, strategies for improving furfural tolerance focus mainly on overexpression of the enzymes that convert furfural to furfuryl alcohol [12,17]. To fill this knowledge gap and at the same time develop strains with strong furfural resistance, genetic determinants of furfural tolerance need to be identified.

Genes associated with furfural tolerance have been identified by comparative analyses of wild-type strains with furfural tolerant mutants generated via random mutation, directed evolution or adaptation strategies. However, the existence of multiple simultaneous mutations in these tolerant strains often complicates the analyses [18]. Therefore, dissecting the functional contribution of each gene towards furfural tolerance remains a significant challenge. RAGE (RNAi-assisted genome evolution) is a recently developed genome engineering method that can continuously improve a desired trait by allowing the sequential introduction of tractable reduction-of-function modifications to the genome [19]. In this study, we used RAGE to select for clones with increased furfural tolerance. The genome-wide RNAi library of *S. cerevisiae* BY4741 was selected for clones with increased furfural tolerance to discover determinants of furfural resistance. Deletion of the gene *SIZ1*, which encodes an E3 SUMO-protein ligase, was found to play an important role in tolerance to furfural and general oxidative stress in *S. cerevisiae*.

## Results

### RAGE screen and isolation of furfural resistant strains

**Figure 1:**
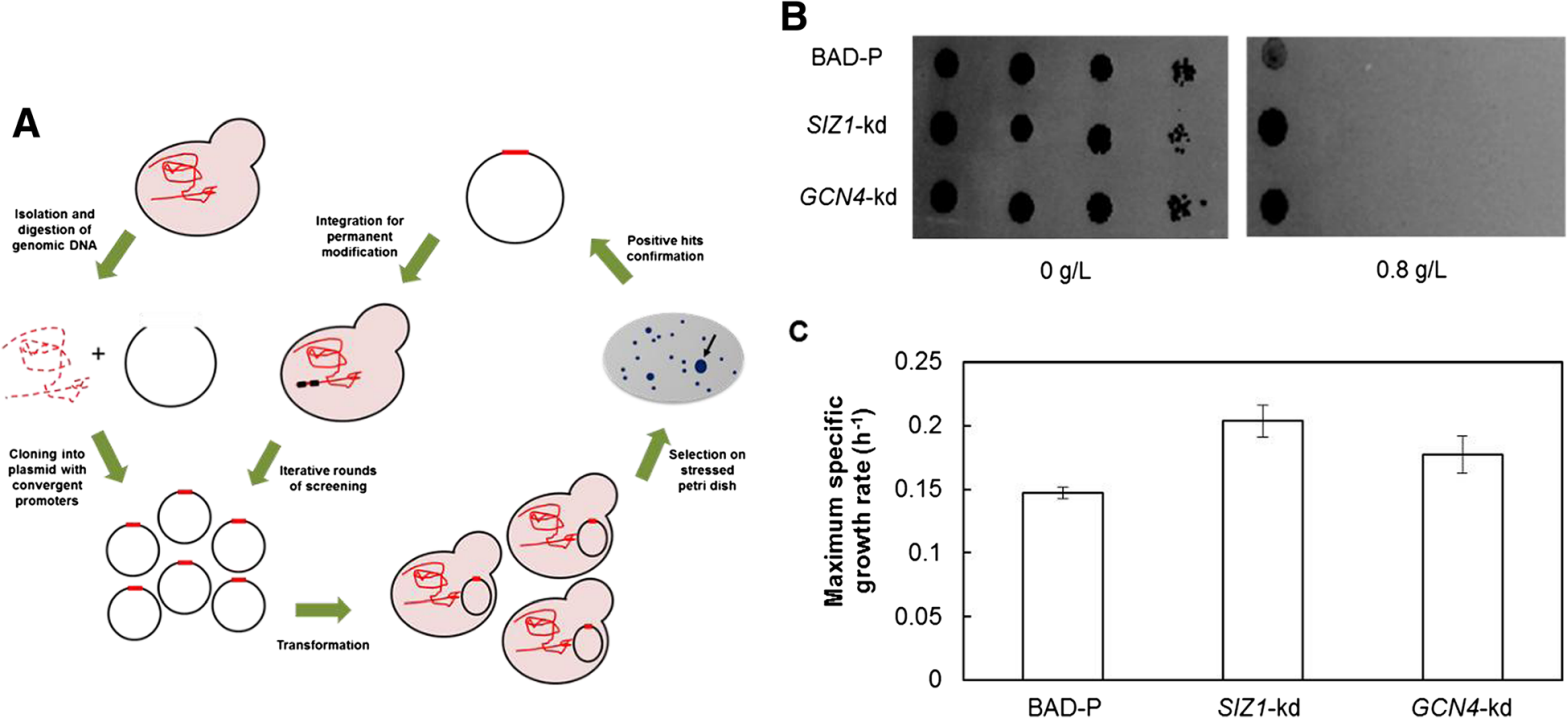
**RNAi-assisted genome evolution (RAGE) screen and isolation of furfural resistant *S. cerevisiae *strains. (A)** Schematic representation of RAGE [19]. **(B)** Isolation of furfural tolerant strains. Ten-fold dilutions of the indicated strains were spotted on synthetic complete (SC) plates containing 0 or 0.8 g/L furfural and incubated at 30°C for 2 and 5 days, respectively. BAD-P is the parent wild-type strain. Strains *SIZ1*-kd and *GCN4*-kd are furfural resistant strains isolated from the RAGE screen. **(C)** Maximum specific growth rates of furfural tolerant strains in the presence of 0.8 g/L furfural. Error bars represent SD (n = 3).

To uncover new genetic determinants of furfural tolerance, we sought to determine if furfural tolerance can be enhanced through reduction of gene function by using RAGE to select for furfural resistant mutants (Figure 1A). Reconstitution of the RNAi machinery in *S. cerevisiae* BY4741 was carried out as previously reported [19] to yield the BAD strain. The genomic DNA derived RNAi library was constructed with additional modifications to prevent self-ligation of vectors and fragments [20]. DNA sequencing of 17 randomly picked plasmids from the RNAi library showed that only one locus was targeted by each RNAi construct (Additional file 1: Figure S1). The number of *Sau3AI* digested fragments (35,837) from *S. cerevisiae* genomic DNA [21] was considered as the number of possible equiprobable variants. With a library size of 3.4 × 10^5^, more than 99% coverage of the yeast genome was achieved [22].

By selecting mutant colonies that grew larger than that of strain BAD-P (strain BAD with plasmid backbone alone) on synthetic complete medium deficient in uracil (SC-URA) plates containing 0.8 g/L furfural, we isolated and confirmed four clones with increased furfural tolerance. Sequencing revealed that three out of the four RNAi constructs from these furfural resistant clones contained the same fragment of the *SIZ1* gene, which encodes an E3 small ubiquitin-like modifier (SUMO)-protein ligase (Additional file 1: Figure S2). The RNAi construct isolated from the fourth clone contained a fragment of the *GCN4* gene encoding a basic leucine zipper transcriptional activator of amino acid biosynthesis (Additional file 1: Figure S3). Strains expressing the RNAi cassettes targeting genes *SIZ1* (*SIZ1*-kd) and *GCN4* (*GCN4*-kd) exhibited significant improvement in furfural tolerance compared to the control BAD-P strain (Figure 1B and C). Notably, *SIZ1*-kd and *GCN4*-kd strains showed no growth advantage over the control strain in the absence of furfural (Additional file 1: Table S3).

### Disruption of *SIZ1 function* increased furfural tolerance in *S. cerevisiae*

To determine if the reduction or loss of *SIZ1* and *GCN4* gene function contributes to furfural resistance, furfural tolerance of the respective knockout mutants was tested. The maximum specific growth rate in the presence of 0.8 g/L furfural of the *siz1Δ* strain was 73% higher than wild type. Increased furfural tolerance was also found in the *siz1Δ* strain when higher furfural concentrations were adopted (Additional file 1: Table S4). Complementation of the *siz1Δ* strain with a plasmid-borne copy of gene *SIZ1* but not with plasmid alone (*siz1Δ-*P) restored furfural sensitivity (Table 1). On the other hand, deletion of *GCN4* did not phenocopy the improved tolerance observed for the *GCN4*-kd strain, suggesting that gene dosage is important for *GCN4* associated furfural tolerance (Table 1). We chose to focus on *SIZ1* as *SIZ1*-kd and *siz1Δ* have a more significant effect on furfural tolerance compared to *GCN4*-kd.

**Table 1:** **Furfural tolerance assay of *siz1Δ *** **mutant and its complementary derivatives**

**Strain**	**Maximum specific growth rate (h**^**-1**^**)**
BAD	0.15 ± 0.00
*siz1Δ*	0.26 ± 0.00
*siz1Δ*-P	0.26 ± 0.01
*siz1Δ*-*SIZ1*	0.19 ± 0.00
*gcn4Δ*	0.16 ± 0.01

Strains were grown in the presence of 0.8 g/L furfural. Results are presented as mean ± SD (n = 3).

To investigate whether increased furfural tolerance via deletion of *SIZ1* is a strain-specific or general attribute, *SIZ1* was also deleted in two other *S. cerevisiae* strains: HZ848 [23] and W303a [24]. Furfural tolerance of these mutants was tested in the presence of 0.8 g/L furfural. Strains HZ848-*siz1Δ* and W303a-*siz1Δ* exhibited 27% and 58% higher maximum specific growth rates respectively, as compared to their respective parent strains (Table 2). These results showed that *SIZ1* was indeed an important determinant for furfural resistance in *S. cerevisiae*.

**Table 2:** **Furfural tolerance assay of *S. cerevisiae siz1Δ *** **mutants in SC medium containing 0.8 g/L furfural**

**Strain**	**Maximum specific growth rate (h**^**-1**^**)**
HZ848	0.15 ± 0.01
HZ848-*siz1Δ*	0.19 ± 0.00
W303a	0.19 ± 0.01
W303a-*siz1Δ*	0.30 ± 0.01

The maximum specific growth rates of different *S. cerevisiae siz1Δ* mutants are statistically significant over their corresponding wild-type (*P* <0.05) as determined by the Student *t*-test. Results are presented as mean ± SD (n = 3).

### Increased rate of furfural reduction and ethanol productivity by *siz1Δ* strain

**Figure 2:**
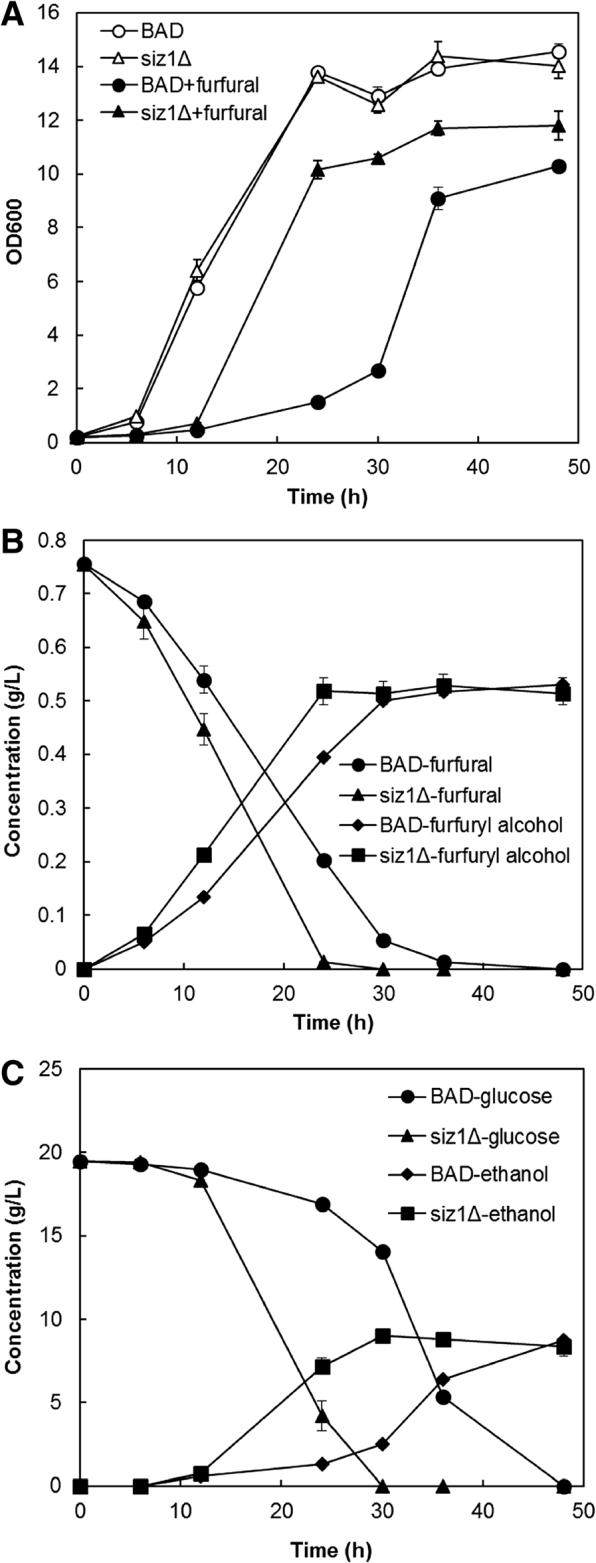
**Growth and metabolite profiles in batch fermentation of parent BAD and *siz1Δ strains*.** Strains were grown in SC medium with 20 g/L glucose in the presence and absence of 0.8 g/L furfural. **(A)** Cell growth as measured by optical density (OD)_600_. **(B)** Furfural consumption and furfuryl alcohol production. **(C)** Glucose consumption and ethanol production. Error bars represent SD of the mean (n = 3).

Having demonstrated that disruption of *SIZ1* gene function greatly increases furfural tolerance, we sought to determine the effect and utility of the enhanced furfural tolerance observed for the *siz1Δ* strain. Batch fermentation containing 20 g/L glucose and 0.8 g/L furfural was conducted using the *siz1Δ* and wild type (BAD) strains. While both control and *siz1Δ* strains experience a delay in entering exponential growth in the presence of furfural, a shorter initial lag was observed in the *siz1Δ* strain (Figure 2A). Finally, furfural was consumed and converted to the less toxic furfuryl alcohol at a rate that was 48% faster in the *siz1Δ* strain compared to that of the wild type (0.031 g/(L · h) versus 0.021 g/(L · h)) (Figure 2B). Strain *siz1Δ* consumed all glucose in 30 h, which was 18 h faster than that of the wild-type strain BAD (Figure 2B and C). As a result, strain *siz1Δ* was able to produce 9.0 g/L ethanol after 30 h, resulting in 275% higher productivity and 254% higher ethanol yield than that observed for strain BAD (Figure 2C, Additional file 1: Table S5). The molar ratios of carbon used for ethanol production were comparable between strain BAD and *∆siz1*, indicating the improved furfural tolerance in strain *∆siz1* was not at the cost of ethanol yield (Additional file 1: Table S5). Overall, these results demonstrate that the increased furfural tolerance observed with disruption of *SIZ1* function was accompanied by faster furfural reduction and this improved trait has clear utility in improving the efficiency of lignocellulose fermentation containing furfural.

### Comparison of *SIZ1* deletion with other previously reported strategies for improving furfural tolerance in *S. cerevisiae*

**Figure 3:**
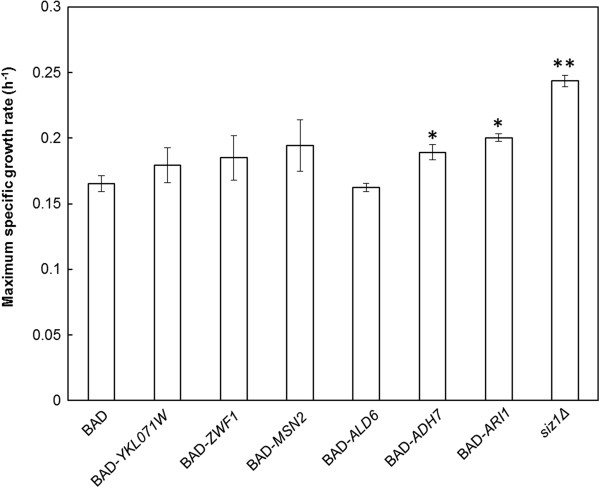
**Maximum specific growth rates to furfural tolerance assay of parent strain BAD and its indicated derivatives in SC medium containing 0.8 g/L furfural.** The Student *t*-test was performed to determine whether the specific growth rates of indicated derivatives was statistically significant over that of the parent strain BAD. *P <0.05; **P <0.01. Error bars represent the SD of the mean (n = 3).

The rapid furfural reduction observed for *siz1Δ* cells is reminiscent of furfural detoxification by enzymes that catalyze aldehyde reduction coupled with cofactors NADPH and/or NADH [13]. Indeed, overexpression of various aldehyde reduction enzymes encoded by genes *YKL071W*, *ALD6*, *ADH7* and *ARI1* have been demonstrated to be strongly associated with furfural resistance in yeast [8,12,17]. In addition, overexpression of glucose-6-phosphate dehydrogenase encoding gene *ZWF1* and transcriptional activator encoding gene *MSN2*, which are involved in regeneration of NAD(P)H and stress response, respectively, have also been confirmed to increase furfural tolerance in *S. cerevisiae*[25,26]. To compare these reported targets with *siz1Δ*-associated furfural tolerance, individual overexpression of each gene was performed in strain BAD. Unexpectedly, only overexpression of gene *ADH7* and *ARI1* resulted in increased furfural tolerance (Figure 3), which may be attributed to the different promoters adopted for over-expression, different growth media tested for furfural tolerance and/or different strains used. Among the engineered strains tested, the *siz1Δ* mutant exhibited the highest maximum specific growth rate in the presence of 0.8 g/L furfural (Figure 3).

### Furfural tolerance is specific to SIZ1p and not to other SUMO E3 ligases

**Figure 4:**
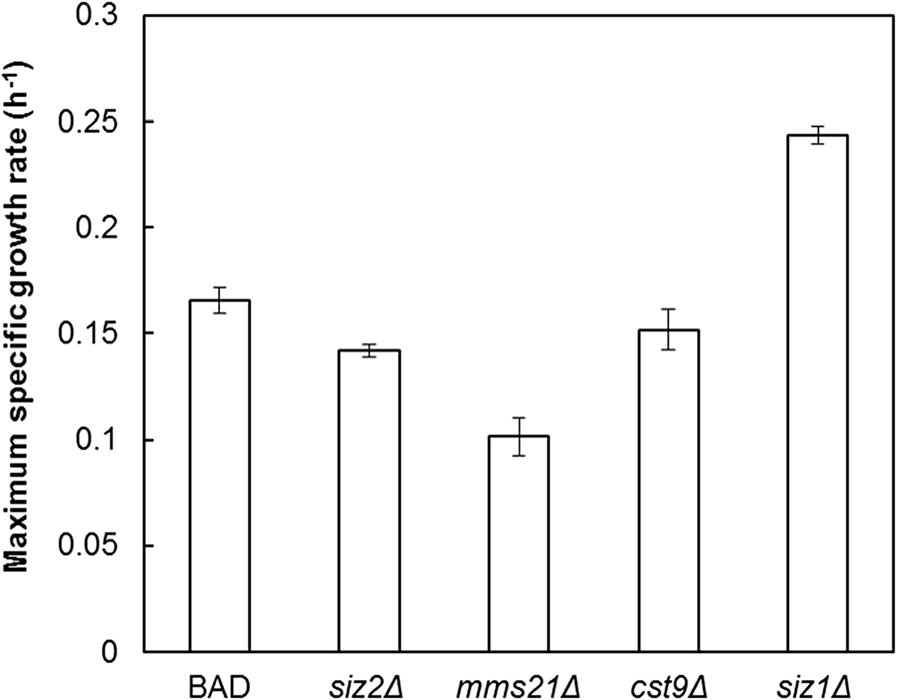
**Furfural tolerance assay of indicated E3 small ubiquitin-like modifier (SUMO)-protein ligase gene deletion mutants in SC medium containing 0.8 g/L furfural.** Error bars represent SD of the mean (n = 3).

Protein sumoylation, an important post-translational modification in various cellular processes, involves the covalent attachment of the SUMO polypeptide to specific lysine residues of target proteins [27]. The E3 SUMO-protein ligase facilitates the transfer of SUMO to the substrate proteins [28]. To investigate whether furfural tolerance is specific to SIZ1p, genes *SIZ2*, *MMS21* and *CST9* that encode for the other three E3 SUMO-protein ligases in yeast [29] were individually deleted but did not affect furfural tolerance of the cells (Figure 4).

**Figure 5:**
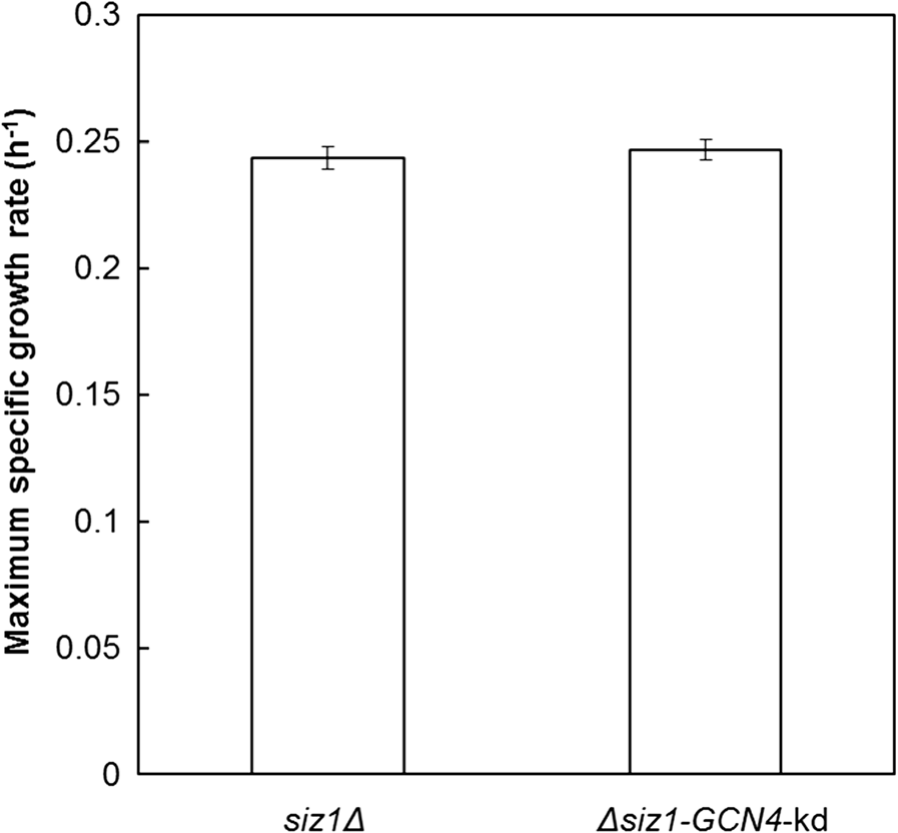
**Furfural tolerance assay of *siz1Δ*strain and its derivatives in SC medium containing 0.8 g/L furfural.** Error bars represent the SD of the mean (n = 3).

As downregulation of gene *GCN4* increased the furfural tolerance of strain BAD (Figure 1), this strategy was evaluated in *siz1Δ* strain. No significant difference in the maximum specific growth rates was found between strain *siz1Δ*-*GCN4*-kd and the *siz1Δ* strain in the presence of 0.8 g/L furfural. This observation suggests that downregulation of *GCN4* may act in the same pathway as *SIZ1* deletion (Figure 5).

### Disruption of *SIZ1* function increases tolerance to oxidative stresses

**Figure 6:**
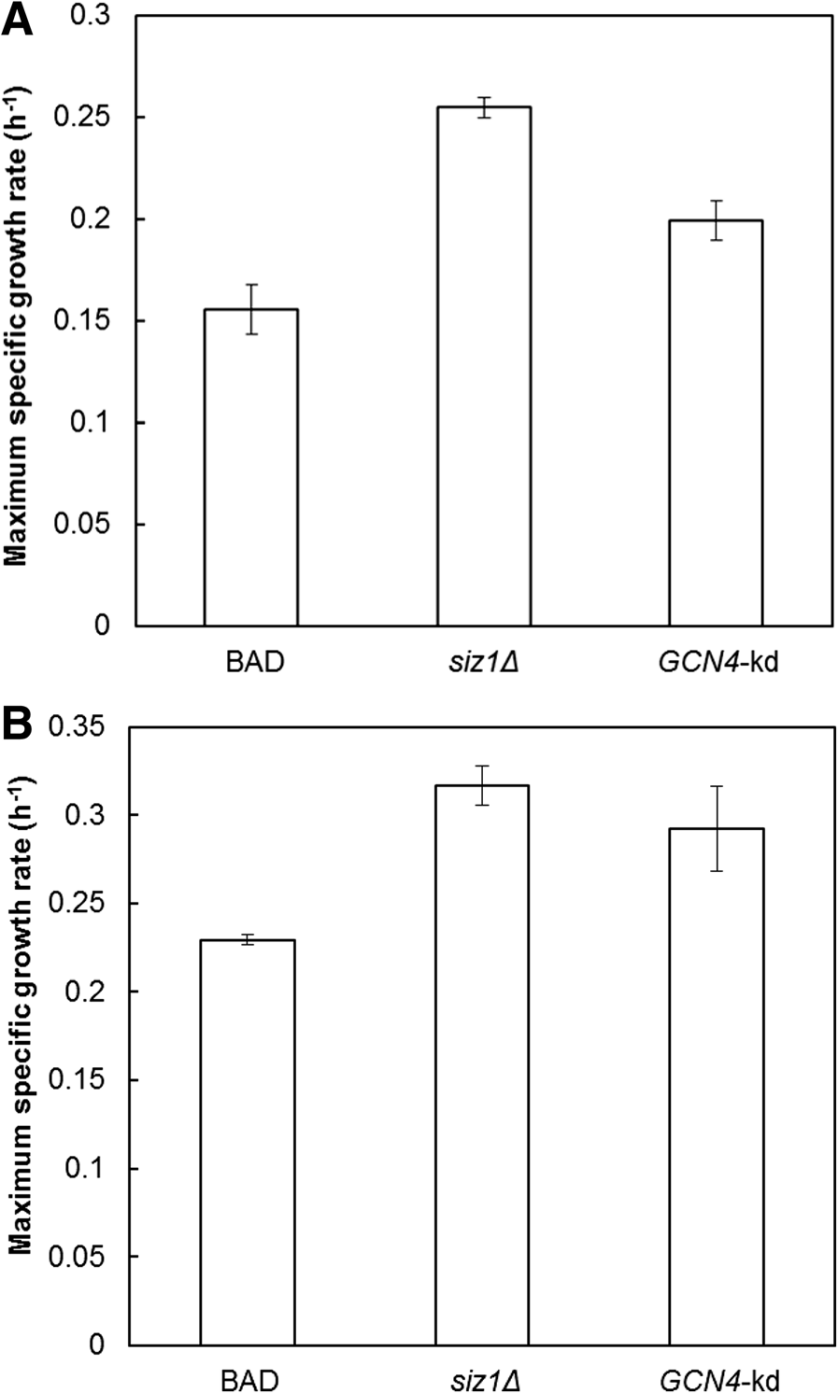
**Tolerance assay of *siz1Δ *strain in SC medium containing (A) 1.26 g/L HMF or (B) 1.72 mg/L menadione.** Error bars represent the SD of the mean (n = 3).

According to a previous study, downregulation of *GCN4* increases tolerance of a furfural-like chemical 5-hydroxymethylfurfural (5-HMF), which is another major inhibitor in lignocellulose hydrolysates that is derived from dehydration of hexoses in lignocellulosic hydrolysates [15,30]. The *siz1Δ* mutant also exhibits higher maximum specific growth rate in the presence of 1.26 g/L 5-HMF compared to the control strain (Figure 6A), while the maximum specific growth rates of both strains were similar in the absence of 5-HMF (Additional file 1: Table S3). Given the higher tolerance achieved by *SIZ1* deletion as compared to downregulation of *GCN4* in the presence of either furfural or 5-HMF, other proteins that are under the SUMO regulation of SIZ1p may also be involved in furfural tolerance besides GCN4p (Figure 1C, Table 1, Figure 6A and Figure 5). Furfural induces accumulation of reactive oxygen species (ROS), the toxicity of which is greatly attenuated in hosts with strong oxidative stress tolerance [10]. To investigate whether *siz1Δ* strain has a detoxification effect on ROS, the oxidative stress tolerance of the *siz1Δ* mutant was further tested in the presence of 1.72 mg/L menadione, which is known to generate ROS *in vivo*[31]. As shown in Figure 6B, the maximum specific growth rate of the *siz1Δ* mutant was 28% higher than that of the wild type. This result indicated that proteins that are related to oxidative stress tolerance may be under the SUMO regulation of *SIZ1* and responsible for furfural tolerance.

## Discussion

Furfural toxicity is a major hurdle in the economical fermentative processes for biofuel and biochemical production using lignocellulosic hydrolysates as substrate [32]. Given the incomplete knowledge on furfural tolerance mechanisms, discovering new targets of furfural resistance would facilitate development of new metabolic engineering strategies for improving furfural tolerance. Whereas significant attention has been paid to the induced genes in furfural tolerance studies, the importance of the repressed genes is often neglected [13]. In this study, RAGE was used for selection of furfural resistant mutants, in order to identify those genes with previously undiscovered roles in furfural tolerance. Genes with downregulation and/or loss of function can be selected out in our case, which distinguishes RAGE from other screening methods (for example, gain-of-function-based screening and *S. cerevisiae* single gene-knockout collection-based screening). In a previous study, RAGE was demonstrated to continuously improve acetic acid tolerance by accumulating reduction-of-function modifications in the genome [19]. In this study, however, no further improvement of furfural tolerance was observed after the second round of selection in the *siz1Δ* strain by RAGE (data not shown).

RNAi cassettes targeting genes *SIZ1* and *GCN4* were recovered during selection for furfural resistance and were shown to increase furfural tolerance (Figure 1). Downregulation of *GCN4* also increased 5-HMF tolerance according to a previous study [15]. GCN4p is a transcriptional activator of gene expression related to amino acid biosynthesis during amino acid starvation in yeast [33,34]. Downregulated expression of *GCN4* could be an efficient means of energy utilization for economic pathway development [15]. However, deletion of *GCN4* did not show increased furfural tolerance in our work, suggesting that an appropriate expression level of amino acid biosynthetic genes may facilitate cell survival under stress challenge.

Replicated discovery of *SIZ1* by RAGE in the presence of furfural, along with the ability of *siz1Δ* to increase furfural tolerance in different *S. cerevisiae* strains, showed the important role of *SIZ1* in furfural resistance (Figure 1, Table 2, Additional file 1: Figure S2). To our knowledge, this is a novel determinant of furfural tolerance. SUMO-modified proteins participate in transcription, nuclear transport, cell cycle, DNA repair and signal transduction [35]. The vast majority of sumoylation in yeast is mediated by SUMO E3 ligase [36]. SIZ1p, together with another SUMO E3 ligase SIZ2p, accounts for 90% of the total sumoylation in yeast [37,38]. As furfural tolerance is *siz1Δ*-dependent (Figure 4), targets that improve furfural tolerance may be exclusively under the SUMO regulation of SIZ1p. Alternatively, these targets may also be under SUMO regulation of other E3 SUMO-protein ligases, but the effect of furfural tolerance from deletion of other E3 SUMO-protein ligases was masked by interactions with other targets which increased susceptibility to furfural.

Downregulation of *GCN4* and deletion of *SIZ1* increased furfural tolerance (Figure 1 and Figure 2). However, no increase of furfural tolerance was found when *GCN4* expression was reduced in the *siz1Δ* strain (Figure 5), indicating these two genes may act in the same pathway. GCN4p stability was demonstrated to be regulated by sumoylation, which occurs after it binds to target promoters and facilitates the subsequent removal of GCN4p from these promoters to ensure accurate transcription of its target genes [39,40]. A possible mechanism for furfural tolerance in the *siz1Δ* strain was speculated upon here. In the *siz1Δ* strain, non-sumoylated GCN4p cannot dissociate from target promoters after recruitment of RNA polymerase II, which may decrease the transcriptional efficiency of the target genes and facilitate cell survival in the presence of furfural.

*SIZ1*-kd or deletion strains exhibited higher furfural tolerance as compared to the *GCN4*-kd strain, indicating more SUMO targets of SIZ1p are likely to be involved in furfural resistance besides GCN4p (Figure 1 and Table 1). A newly identified SUMO substrate of SIZ1p is the NADHX dehydratase YKL151Cp, which converts (S)-NADHX to NADH [41,42]. More reducing power for furfural detoxification may be generated through this reaction. In addition, the *siz1Δ* strain also exhibited increased oxidative stress tolerance, suggesting that determinants of oxidative stress tolerance may be protein substrates of SIZ1p responsible for furfural tolerance (Figure 6B). A total of 159 proteins were identified to be sumoylated in a proteomics study [40], among which the 6-phosphogluconate dehydrogenase GND1p, the basic leucine zipper transcription factor SKO1p and the redoxin peroxidase TSA1p are implicated in oxidative stress response in yeast [43-45]. These candidates could be possible determinants of furfural resistance under SUMO regulation of SIZ1p. Identification of protein substrates that are differentially sumoylated in wild type and *siz1Δ* cells in the presence of furfural will further reveal the detailed molecular mechanism of furfural resistance in the mutant.

## Conclusions

In this study, RNAi knockdown of genes *SIZ1* and *GCN4* was demonstrated to improve furfural tolerance in *S. cerevisiae*. The *siz1Δ* mutant was further found to exhibit superior performance with cell growth, glucose consumption, furfural consumption and ethanol productivity as compared to the parent strain, while the *gcn4Δ* strain did not exhibit improved furfural tolerance. Deletion of *SIZ1* also resulted in higher furfural tolerance in different *S. cerevisiae* strains, indicating *SIZ1* deletion may play an important role in furfural resistance in *S. cerevisiae* strains. To our knowledge, this is a novel determinant of furfural resistance. Preliminary exploration of furfural tolerance in the *siz1Δ* mutant showed that the proteins responsible for furfural tolerance, among which GCN4p is a possible candidate, may be exclusively under the SUMO regulation by SIZ1p. Besides furfural tolerance, the *siz1Δ* mutant also exhibited tolerance towards oxidative stress, suggesting that proteins that are related to oxidative stress tolerance may be under the SUMO regulation of SIZ1p and responsible for furfural tolerance. These findings provide valuable insights into the engineering of furfural resistant microbes for efficient lignocellulose-based fermentation.

## Methods

### Strains and growth media

The strains and plasmids used in this study are listed in Table 3. Cells were grown in liquid SC [46], SC-URA or synthetic complete medium deficient in leucine (SC-LEU) supplemented with 20 g/L glucose as the carbon source or on solid 1% yeast extract, 2% peptone, 0.01% adenine hemisulfate, 2% glucose and 2% agar (YPAD) medium unless otherwise noted. The initial pH value of SC medium was adjusted to 5.6 using 12 M NaOH.

**Table 3:** Strains and plasmids used in this study

**Strains or plasmids**	**Characteristics**	**Reference or source**
Strains		
*S. cerevisiae*		
BY4741	*MATa his3Δ0 leu2Δ0 met15Δ0 ura3Δ0*	[47]
BAD	BY4741/*δ::*TEF1p-ago1-TPI1p-dcr1	This study
BAD-P	BAD/pRS416-TTrcx	This study
*SIZ1*-kd	BAD/pRS416-TTrcx-siz1	This study
*GCN4*-kd	BAD/pRS416-TTrcx-gcn4	This study
*siz1Δ*	BAD/*siz1Δ*::leu2	This study
*siz1Δ*-P	*siz1Δ*/pRS416e	This study
*siz1Δ*-*SIZ1*	*siz1Δ*/pRS416e-siz1	This study
*gcn4Δ*	BAD/*gcn4Δ*::leu2	This study
*siz2Δ*	BAD/*siz2Δ*::leu2	This study
*mms21Δ*	BAD/*siz1Δ*::leu2	This study
*cst9Δ*	BAD/*cst9Δ*::leu2	This study
BAD-*YKL071W*	BAD/pRS416e-ykl071w	This study
BAD-*ZWF1*	BAD/pRS416e-zwf1	This study
BAD-*MSN2*	BAD/pRS416e-msn2	This study
BAD-*ALD6*	BAD/pRS416e-ald6	This study
BAD-*ADH7*	BAD/pRS416e-adh7	This study
BAD-*ARI1*	BAD/pRS416e-ari1	This study
*siz1Δ*-*GCN4*-kd	*siz1Δ*/pRS416-TTrcx-gcn4	This study
HZ848	*MATα, ade2-1, Δura3, his3-11, 15, trp1-1, leu2-3, 112, and can1-100*	[23]
HZ848-*siz1Δ*	HZ848/*siz1Δ*::ura3	This study
W303a	*MATa; ura3-1; trp1Δ 2; leu2-3,112; his3-11,15; ade2-1; can1-100*	[24]
W303a-*siz1Δ*	W303a/*siz1Δ*::hygromycin B	This study
*E. coli*		
DH5α	General cloning host	Takara
WM1788	Cloning host	Provided by Professor William Metcalf
Plasmids		
pRS416	Yeast centromere with URA3 marker	[48]
pRS425-TEF1p-PmeI-PGK1t	Yeast gene expression vector	[19]
pRS416e	Derived from pRS416, with *TEF1* promoter and *PGK1* terminator added	This study
pRS-delta-KanMX-LoxP-TEF1p-AGO1-PGK1t-TPI1p-DCR1-GPD1t	Helper plasmid for integration of *S. castellii* RNAi pathway into delta-site	[19]
pRS416-TTrc	Derived from pRS416, with convergent promoters to produce dsRNA	[19]
pRS416-TTrcx	Derived from pRS416-TTrc, with *Xho*I restriction recognition sequence instead of *BamH*I	This study
pRS416-TTrcx-siz1	Derived from pRS416-TTrcx, with gene *SIZ1* fragment added	This study
pRS416-TTrcx-gcn4	Derived from pRS416-TTrcx, with gene *GCN4* fragment added	This study
pRS415	Yeast centromere with LEU2 marker	[48]
pUG6	The loxP-KanMX-loxP disruption module	Euroscarf
pUG72	The loxP-URA3-loxP disruption module	Euroscarf
pLHCX	Template for amplification of hygromycin B resistance gene	Clontech
pXZ5	Derived from pUG72, with hygromycin B resistance gene expression cassette instead of ura3	This study
pRS416e-siz1	Derived from pRS416e, with *SIZ1* gene cassette added	This study
pRS416e-ykl071w	Derived from pRS416e, with *YKL071W* gene cassette added	This study
pRS416e-zwf1	Derived from pRS416e, with *ZWF1* gene cassette added	This study
pRS416e-msn2	Derived from pRS416e, with *MSN2* gene cassette added	This study
pRS416e-ald6	Derived from pRS416e, with *ALD6* gene cassette added	This study
pRS416e-adh7	Derived from pRS416e, with *ADH7* gene cassette added	This study
pRS416e-ari1	Derived from pRS416e, with *ARI1* gene cassette added	This study

### Construction of plasmids, genome-wide RNAi library and reconstitution of RNAi machinery in *S. cerevisiae* BY4741

The primers used in this study are listed in Additional file 1: Table S1. Plasmid constructions are summarized in Additional file 1: Table S2. All plasmid construction was performed by In-fusion HD cloning (Clontech Laboratories, Inc., Mountain View, CA, USA) following the manufacturer’s instructions, or by the DNA assembler method [23]. Construction of the genomic library of *S. cerevisiae* BY4741 was carried out as previously described [19] with modifications to prevent self-ligation of vectors and fragments [20]. Finally, a library size of 2.6 × 10^6^ transformants was obtained, while the control reaction with only linearized plasmid gave 4 × 10^4^ transformants. The plasmid library was isolated from an overnight *E. coli* culture.

### DNA transformation of *S. cerevisiae* strains

DNA transformation of *S. cerevisiae* strains was carried out using the method developed by Gietz and Schiestl [49].

### RAGE screen for increased furfural tolerance

The RNAi library (20 μg) or control plasmid pRS416-TTrcx was transformed into the BAD strain harboring the RNAi machinery. A library size of 3.4 × 10^5^ was achieved, ensuring >99% coverage of the yeast genome [22]. Following transformation, yeast cells were recovered in 1 mL YPAD medium for 4 h, washed with ddH_2_O and plated onto solid SC-URA medium containing 0.8 g/L furfural. The library and control plates were incubated at 30°C for 3 to 5 days. Thirty-three colonies of sizes bigger than the largest colonies on the control plates were picked from the library plates into SC-URA liquid medium. The growth performance of the selected colonies and control strain were compared in the presence of 0.8 g/L furfural. The initial OD_600_ for all the strains was 0.2, and the growth rate was measured after 24 h. The RNAi plasmids from the top 14 strains with OD_600_ values at least 20% higher than the control strain were isolated and amplified in *E. coli*. The selected plasmids were then individually retransformed, of which four were able to retain the enhanced furfural tolerance in a fresh genetic background with three biological replicates. The four plasmids were sequenced with the primer pRS416-TTrc-S (Additional file 1: Table S1).

### Spot assay

Yeast cells in the stationary phase were transferred into 5 mL of SC media in a 15-mL round-bottom Falcon tube at an initial OD_600_ of 0.2 and grown to an OD_600_ of 0.7 (30°C, 250 rpm). The cells were serially diluted 10-fold with sterile water and 5 μl of each dilution was spotted onto furfural-free SC agar (control) and SC agar medium containing 0.8 g/L furfural. The plates were incubated at 30°C for 2 to 5 days.

### Tolerance assay - calculation of maximum specific growth rates

Maximum specific growth rate was used as an indicator for the cellular tolerance towards various inhibitors [12,50,51]. For calculating the maximum specific growth rates of *S. cerevisiae* strains, stationary-phase cells grown in SC medium were transferred into 5 mL of SC medium containing a specific inhibitor (0.8 g/L furfural, 1.2 g/L furfural, 2.0 g/L furfural, 1.26 g/L HMF or 1.72 mg/L menadione) in a 15-mL round-bottom Falcon tube (30°C, 250 rpm). The initial OD_600_ was 0.2. The maximum specific growth rate was determined from the maximum slope of the OD_600_ values over time.

### Fermentation

Batch fermentations were carried out as follows: a single colony grown on a YPAD plate was inoculated into 3 mL of SC medium containing 20 g/L glucose in a 15-mL round-bottom Falcon tube and grown until saturation (30°C, 250 rpm). About 400 μL of the stationary-phase cells were transferred into 25 mL of fresh SC media containing 0.8 g/L furfural in 250 mL non-baffled shake flasks. Cells were grown under oxygen-limited conditions (30°C, 100 rpm) as previously reported [46]. The initial OD_600_ was 0.2.

### HPLC analysis

The samples were centrifuged and the supernatants were diluted five to ten times before HPLC analysis. An Agilent 1100 series HPLC (Agilent Technologies, Palo Alto, CA, USA) coupled with an Agilent ZORBAX 80A Extend-C18 column was used for detection of furfural and furfuryl alcohol. HPLC parameters were as follows: solvent A, water; solvent B, acetonitrile; 5% B for 15 minutes, then 100% B for 5 minutes, followed by 5% B for 5 minutes; flow rate 1 mL/minute; detection by UV spectroscopy at 277 nm (furfural) or 210 nm (furfuryl alcohol). Under such conditions, furfural and furfuryl alcohol were eluted at 6.7 minutes and 5.6 minutes, respectively. An HPLC system equipped with a refractive index detector (Shimadzu Scientific Instruments, Columbia, MD, USA) was used to analyze the concentrations of glucose and ethanol in the broth. To separate glucose and ethanol, an HPX-87H column (BioRad, Hercules, CA, USA) was used as described [46].

## Abbreviations

5-HMF: 5-hydroxymethylfurfural; OD: optical density; NAPDH: reduced nicotinamide adenine dinucleotide phosphate; RAGE: RNAi-assisted genome evolution; RNAi: RNA interference; ROS: reactive oxygen species; SC: synthetic complete; SC-LEU: synthetic complete medium deficient in leucine; SC-URA: synthetic complete medium deficient in uracil; SUMO: small ubiquitin-like modifier.

## Competing interests

The authors declare that they have no competing interests.

## Authors’ contributions

All the experiments were performed by HX. Both authors contributed to designing the experiments, writing the manuscript and have approved the final manuscript

## Supplementary Material

Additional file 1: Figure S1 Sequencing of 17 randomly picked plasmids from the RNAi library. Locations have been mapped to the *S. cerevisiae* genome. Each column represents one chromosome, the height of which is proportional to the size of the indicated chromosome. Each horizontal bar indicates the location of a fragment. **Figure S2.** Sequencing result of pRS416-TTrcx-siz1, which contains a fragment of gene *SIZ1* (underlined). **Figure S3.** Sequencing result of pRS416-TTrcx-gcn4, which contains a fragment of gene *GCN4* (underlined). **Table S1.** Primers used in this study. **Table S2.** Construction of plasmids. **Table S3.** Maximum specific growth rates of strain BAD and its derivatives cultured in SC medium containing 20 g/L glucose. **Table S4.** Maximum specific growth rates of strain BAD and its derivatives cultured in SC medium containing different concentrations of furfural. **Table S5.** Fermentation parameters and estimation of carbon balance in strain BAD and *siz1Δ* after 30 h in SC medium containing 20 g/L glucose and 0.8 g/L furfural. Click here for file
