# Supplementary material for: Genome-wide RNAi screen reveals the E3 SUMO-protein ligase gene SIZ1 as a novel determinant of furfural tolerance in Saccharomyces cerevisiae
Source: Biotechnol Biofuels. 2014 May 23;7:78. doi: 10.1186/1754-6834-7-78 (PMC4045865; doi:10.1186/1754-6834-7-78)
Supplement: Additional file 1: Figure S1 — Sequencing of 17 randomly picked plasmids from the RNAi library. Locations have been mapped to the S. cerevisiae genome. Each column represents one chromosome, the height of which is proportional to the size of the indicated chromosome. Each horizontal bar indicates the location of a fragment. Figure S2. Sequencing result of pRS416-TTrcx-siz1, which contains a fragment of gene SIZ1 (underlined). Figure S3. Sequencing result of pRS416-TTrcx-gcn4, which contains a fragment of gene GCN4 (underlined). Table S1. Primers used in this study. Table S2. Construction of plasmids. Table S3. Maximum specific growth rates of strain BAD and its derivatives cultured in SC medium containing 20 g/L glucose. Table S4. Maximum specific growth rates of strain BAD and its derivatives cultured in SC medium containing different concentrations of furfural. Table S5. Fermentation parameters and estimation of carbon balance in strain BAD and siz1Δ after 30 h in SC medium containing 20 g/L glucose and 0.8 g/L furfural. [file 1754-6834-7-78-S1.docx]

# Additional file 1

### Fig. S1. Sequencing of 17 randomly picked plasmids from the RNAi library. Locations have been mapped to the *S. cerevisiae* genome. Each column represents one chromosome, the height of which is proportional to the size of the indicated chromosome. Each horizontal bar indicates the location of a fragment.


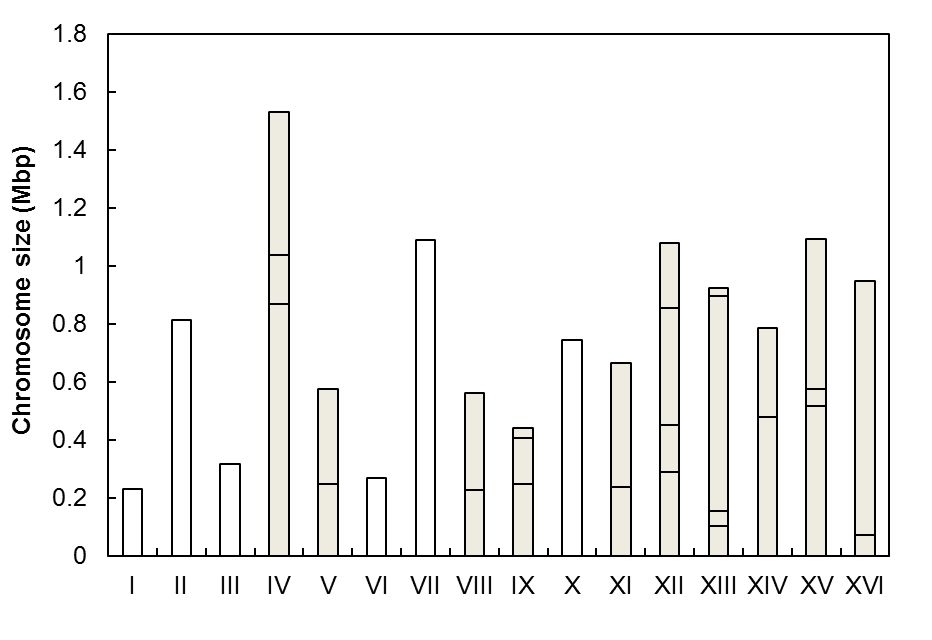


### Fig. S2. Sequencing result of pRS416-TTrcx-siz1, which contains a fragment of gene *SIZ1* (underlined).

AAACTTTAATTACAAACTCGATCCGCATACTGAAGTACCTTTTTCTGGGCTTCTACTACCATCATTGCTGTCACTGTTCGCTGTCATCATCGTCTTCCAATATGGCTGTCCACTTACCATCAGAAGTAAGTTCTACCTGTTCAACGTTTTTTTGACAGTTCTGTAAAATGTCATCAACAAATTCGGAAATTGCTAAATTTTCCAAAGCTATATCAATTTGACATACTGGGCATTGCCACGTAGGAATTTGTAGTTGGGAGTGTAGGAACCATAATGCATCAAAACATTGCAGATGCTTACAATTTATTGATTTTGAAGGGTATTTCATTCTTGTGTACGAAATTGGACATTGCAGACTCATGATAGTAGATGTGGTAGTCAAGCCCATTTCTTCATCCTCCCGAAGTGTTTTTTTCAAGTAAAGTAACGTGGCTTGTTTAATAATTTTTGGATGCTGTAATACTTTTTCCAGGAGTTGCTCCGGAGTGATCGAGTTAGTTTATGTATGTGTTTTTTGTAGTTATAGATTTAAGCAAGAAAAGAATACAAACAAAAAATTGAAAAAGATTGATTTAGAATTAAAAAGAAAAATATTTACGTAAGAAGGGAAAATAGTAAATGTTGCAAGTTCACTAAACTCCTAAATTATGCTGCCCTTTATATTCCCTGTTACAGCAGCCGAGCCAAAGGTATATAGGCTCCTTTGCATTAGCATGCGTAACAAACCACCTGTCAGTTTCAACCGAGGTGGTATCCGAGAGAATTGTGTGATTGCTTTAATTAATTTCGGAGAATCTCACATGCCACTGAAGATTAAAAACTGGATGCCAGAAAAGGGGTGTCCAGGTGTAACATCAATAGAGGAAGCTGAAAAAGTCTTAGAACGGGTAATCTTCCACCAACCTGATGGGTTCCTAGATATAATTGAATTGAATTGAAATCGATAGATCAATTTTTTTTCTTTTCTCTTTCCCCATCCCTTTACCCTAAAAATAATAGCTTTATTTTATTTTTTGAATATTTTTTATTTATATACCGTATATATAAGACTATTATTTATTCTTTAATGATTATAAAGAT

### Fig. S3. Sequencing result of pRS416-TTrcx-gcn4, which contains a fragment of gene *GCN4* (underlined).

ACAAACTCGATCCAGTCTCGATTCGTCATCCTTTCCAACATGATGTGACTTCTTAACGACTGAATTTGGTTTCTTAACCTTTCTTGTTTGAGTCAGTTTAGCATCTTCTAGAACAGGAGTGGGTAAGAATGAAGTTGTCGAGACTTCCAGATTGGATGGTACCAGAGAAACTTCTTCAGTGGATTCAATTGCCTTATCAGCCAATGAAACATCGTCAGTGGTAACTGGAATGTCATTGTCAAACAAGGATGTCCATTCTTTAGAGTTGTCTTCTAGGTTTTCATACTCAAACATTGGAGTTGAATCAGTGCTTGACGAAAAGAAAGATTCCACTACAGCGTCATCTAGCTCCGGAATTGGCAAAACGGTCTTGGCATCAGGTGCAGTTGCCGTTTGTGGAAGAGCAAAATCAAAATCAAGGTTCGAAGGGGTATCCTGTTTGATAATTGGATCGAGTTAGTTTATGTATGTGTTTTTTGTAGTTATAGATTTAAGCAAGAAAAGAATACAAACAAAAAATTGAAAAAGATTGATTTAGAATTAAAAAGAAAAATATTTACGTAAGAAGGGAAAATAGTAAATGTTGCAAGTTCACTAAACTCCTAAATTATGCTGCCCTTTATATTCCCTGTTACAGCAGCCGAGCCAAAGGTATATAGGCTCCTTTGCATTAGCATGCGTAACAAACCACCTGTCAGTTTCAACCGAGGTGGTATCCGAGAGAATTGTGTGATTGCTTTAATTAATTTCGGAGAATCTCACATGCCACTGAAGATTAAAAACTGGATGCCAGAAAAGGGGTGTCCAGGTGTAACATCAATAGAGGAAGCTGAAAAGTCTTAGAACGGGTAATCTTCCACCAACCTGATGGGTTCCTAGATATAATTGAATTGAATTGAAATCGATAGATCAATTTTTTTCTTTTCTCTTTCCCCATCCTTTACGCTAAAATAATAGTTTATTTTATTTTTTGAATATTTTTTATTTATATACGTATATATAGACTATTATTTATCTTTTAATGATTATTAAGATTTTTATTAAAAAAAAATTCCCTC

### Table S1. Primers used in this study.

| **Primer** | **Sequence (5’->3’)** | **Description** |
| --- | --- | --- |
| pRS416-TTrc-XhoI-up | atctaagttttaattacaaactcgagttagtttatgtatgtgtttt | Forward primer for construction of pRS416-TTrcx |
|  |  |  |
| pRS416-TTrc-ClaI-dn | GAAAAGAAAAAAATTGATCT | Reverse primer for construction of pRS416-TTrcx |
|  |  |  |
| pRS416-TTrc-S | ttttacttcttgctcattag | Forward sequencing primer |
|  |  |  |
| pXZ5-HXT7p-up | tataatgtatgctatacgaagttattaggtctagagatctacttctcgtaggaacaattt | Forward primer for *HXT7* promoter |
|  |  |  |
| pXZ5-HXT7t-dn | CAGACGTCGCGGTGAGTTCAGGCTTTCCGGATCTATCCATTTTTTGATTAAAATTAAAAA | Reverse primer for *HXT7* promoter |
|  |  |  |
| pXZ5-hyg-up | aaacacaaaaacaaaaagtttttttaattttaatcaaaaaatggatagatccggaaagcc | Forward primer for hygromycin B resistance gene |
|  |  |  |
| pXZ5-hyg-dn | AATACTCATTAAAAAACTATATCAATTAATTTGAATTAACCTATTCCTTTGCCCTCGGAC | Reverse primer for hygromycin B resistance gene |
|  |  |  |
| pXZ5-FBA1t-up | aaaccgacgccccagcactcgtccgagggcaaaggaataggttaattcaaattaattgat | Forward primer for *FBA1* terminator |
|  |  |  |
| pXZ5-FBA1t-dn | ATACATTATACGAAGTTATATTAAGGGTTCTCGAGAGCTCAAAGATGAGCTAGGCTTTTG | Reverse primer for *FBA1* terminator |
|  |  |  |
| siz1-leu-up | caactcaaacagttgagtgttccatatacattctgtttcaatgtctgcccctatgtctgc | Forward primer for *SIZ1* deletion cassette in *S. cerevisiae* BY4741 |
|  |  |  |
| siz1-leu-dn | TGAAAGAGCTGGACGGAACCGTCCAATTTTAGCCTCGTTTTTAAGCAAGGATTTTCTTAA | Reverse primer for *SIZ1* deletion cassette in *S. cerevisiae* BY4741 |
|  |  |  |
| pRS-TEF1p For | taaaacgacggccagtgagcgcgcgtaatacgactcacagcaacaggcgcgttggac | Forward primer for *TEF1* promoter |
|  |  |  |
| PGK1t-pRS Rev | GATTACGCCAAGCGCGCAATTAACCCTCACTAAAGGGAACCAGGAAGAATACACTATAC | Reverse primer for *PGK1* terminator |
|  |  |  |
| pRS416e-siz1-up | ttaattacaaagtttatgataaatttagaggatta | Forward primer for *SIZ1* gene |
|  |  |  |
| pRS416e-siz1-dn | TTCAATTCAATGTTTTTAACCACTGTTGTATTTCT | Reverse primer for *SIZ1* gene |
|  |  |  |
| siz1-del-up | ccaactcaaacagttgagtgttccatatacattctgtttcaCAGCTGAAGCTTCGTACGC | Forward primer for *SIZ1* deletion cassette in *S. cerevisiae* HZ848 and W303a |
|  |  |  |
| siz1-del-dn | AAAGAGCTGGACGGAACCGTCCAATTTTAGCCTCGTTTGCATAGGCCACTAGTGGATCTG | Reverse primer for *SIZ1* deletion cassette in *S. cerevisiae* HZ848 and W303a |
|  |  |  |
| gcn4-leu-up | caatttgtctgctcaagaaaataaattaaatacaaataaaatgtctgcccctatgtctgc | Forward primer for *GCN4* deletion cassette |
|  |  |  |
| gcn4-leu-dn | GAGAATGAAATAAAAAATATAAAATAAAAGGTAAATGAAATTAAGCAAGGATTTTCTTAA | Reverse primer for *GCN4* deletion cassette |
|  |  |  |
| siz2-leu-up | tacactgataatcaagaaacgtataagggaaaagagcacgatgtctgcccctatgtctgc | Forward primer for *SIZ2* deletion cassette |
|  |  |  |
| siz2-leu-dn | AGAATACAATCGGAAAGGAAAGAAATCAAAAGACGGTTAATTAAGCAAGGATTTTCTTAA | Reverse primer for *SIZ2* deletion cassette |
|  |  |  |
| mms21-leu-up | aaccaaggcaagactatataaaaaaagaataactttaaaaatgtctgcccctatgtctgc | Forward primer for *MMS21* deletion cassette |
|  |  |  |
| mms21-leu-dn | GGGCCGAAGGGCTCGGATAAGAGAAACAATAATTTTGTTTTTAAGCAAGGATTTTCTTAA | Reverse primer for *MMS21* deletion cassette |
|  |  |  |
| cst9-leu-up | cgtctgtgaagttgacgctttgtgcggcggccaacaagggatgtctgcccctatgtctgc | Forward primer *CST9* deletion cassette |
|  |  |  |
| cst9-leu-dn | TCTGAAGGCTGTTTTCGTCACGGGGAATCCTTACACCTATTTAAGCAAGGATTTTCTTAA | Reverse primer for *CST9* deletion cassette |
|  |  |  |
| ykl071w-up | ttaattacaaagtttatgaatacttcatcaagaat | Forward primer for *YKL071W* gene |
|  |  |  |
| ykl071w-dn | TTCAATTCAATGTTTCTAAAAGACGCCTTCGCTGC | Reverse primer for *YKL071W* gene |
|  |  |  |
| zwf1-up | ttaattacaaagtttatgagtgaaggccccgtcaa | Forward primer for *ZWF1* gene |
|  |  |  |
| zwf1-dn | TTCAATTCAATGTTTCTAATTATCCTTCGTATCTT | Reverse primer for *ZWF1* gene |
|  |  |  |
| msn2-up | ttaattacaaagtttatgacggtcgaccatgattt | Forward primer for *MSN2* gene |
|  |  |  |
| msn2-dn | TTCAATTCAATGTTTTTAAATGTCTCCATGTTTTT | Reverse primer for *MSN2* gene |
|  |  |  |
| ald6-up | ttaattacaaagtttatgactaagctacactttga | Forward primer for *ALD6* gene |
|  |  |  |
| ald6-dn | TTCAATTCAATGTTTTTACAACTTAATTCTGACAG | Reverse primer for *ALD6* gene |
|  |  |  |
| adh7-up | ttaattacaaagtttatgctttacccagaaaaatt | Forward primer for *ADH7* gene |
|  |  |  |
| adh7-dn | TTCAATTCAATGTTTCTATTTATGGAATTTCTTAT | Reverse primer for *ADH7* gene |
|  |  |  |
| ari1-up | ttaattacaaagtttatgactactgataccactgt | Forward primer for *ARI1* gene |
|  |  |  |
| ari1-dn | TTCAATTCAATGTTTTTAGGCTTCATTTTGAACTT | Reverse primer for *ARI1* gene |

### Table S2. Construction of plasmids.

| Plasmid | Primers for PCR | Template for PCR | Vector/linearization enzymes | Cloning method |
| --- | --- | --- | --- | --- |
| pRS416-TTrcx | pRS416-TTrc-XhoI-up/ pRS416-TTrc-ClaI-dn | pRS416-TTrc | pRS416-TTrc/ XhoI and ClaI | In-fusion HD cloning |
|  |  |  |  |  |
| pXZ5 | pXZ5-HXT7p-up/ pXZ5-HXT7p-dn, pXZ5-hyg-up/ pXZ5-hyg-dn, pXZ5-FBA1t-up/ pXZ5-FBA1t-dn | Genomic DNA of *S. cerevisiae* BY4741 and plasmid pLHCX | pUG6/ BglII and SacI | DNA assembler [[1](#_ENREF_1)] |
|  |  |  |  |  |
| pRS416e | pRS-TEF1p For/ PGK1t-pRS Rev | pRS425-TEF1p-PmeI-PGK1t | pRS416/ HindIII and EcoRI | DNA assembler [[1](#_ENREF_1)] |
|  |  |  |  |  |
| pRS416e-siz1 | pRS416e-siz1-up/ pRS416e-siz1-dn | Genomic DNA of *S. cerevisiae* BY4741 | pRS416e/ PmeI | In-fusion HD cloning |
|  |  |  |  |  |
| pRS416e-ykl071w | ykl071w-up/ ykl071w-dn | Genomic DNA of *S. cerevisiae* BY4741 | pRS416e/ PmeI | In-fusion HD cloning |
|  |  |  |  |  |
| pRS416e-zwf1 | zwf1-up/ zwf1-dn | Genomic DNA of *S. cerevisiae* BY4741 | pRS416e/ PmeI | In-fusion HD cloning |
|  |  |  |  |  |
| pRS416e-msn2 | msn2-up/ msn2-dn | Genomic DNA of *S. cerevisiae* BY4741 | pRS416e/ PmeI | In-fusion HD cloning |
|  |  |  |  |  |
| pRS416e-ald6 | ald6-up/  ald6-dn | Genomic DNA of *S. cerevisiae* BY4741 | pRS416e/ PmeI | In-fusion HD cloning |
|  |  |  |  |  |
| pRS416e-adh7 | adh7-up/ adh7-dn | Genomic DNA of *S. cerevisiae* BY4741 | pRS416e/ PmeI | In-fusion HD cloning |
|  |  |  |  |  |
| pRS416e-ari1 | ari1-up/  ari1-dn | Genomic DNA of *S. cerevisiae* BY4741 | pRS416e/ PmeI | In-fusion HD cloning |

## Table S3. Maximum specific growth rates of strain BAD and its derivatives cultured in SC medium containing 20 g/L glucose. Error bars represent the standard deviation of the mean (n=3).

| Strain | Maximum specific growth rate (h^-1^) |
| --- | --- |
| BAD | 0.33 ± 0.03 |
| *siz1Δ* | 0.33 ± 0.02 |
| *gcn4Δ* | 0.34 ± 0.02 |
| *siz1Δ*-*GCN4*-kd | 0.35 ± 0.03 |
| BAD-P | 0.37 ± 0.01 |
| *SIZ1*-kd | 0.39 ± 0.01 |
| *GCN4*-kd | 0.39 ± 0.04 |

**Table S4.** **Maximum specific growth rates of strain BAD and its derivatives cultured in SC medium containing different concentrations of furfural.** Error bars represent the standard deviation of the mean (n=3). For those mutants with no obvious cell growth observed after 72 h incubation, the maximum specific growth rates are represented by dash.

| Furfural concentration (g/L) | Strain | Maximum specific growth rate (h^-1^) |
| --- | --- | --- |
| 1.2 | BAD | 0.16 ± 0.00 |
|  | *siz1Δ* | 0.18 ± 0.00 |
|  | *gcn4Δ* | 0.16 ± 0.01 |
|  | *SIZ1*-kd | 0.18 ± 0.03 |
|  | *GCN4*-kd | 0.18 ± 0.02 |
| 2.0 | BAD | - |
|  | *siz1Δ* | 0.15 ± 0.01 |
|  | *gcn4Δ* | - |
|  | *SIZ1*-kd | 0.17 ± 0.01 |
|  | *GCN4*-kd | - |

**Table S5. Fermentation parameters and estimation of carbon balance in strain BAD and *siz1Δ* after 30 h in SC medium containing 20 g/L glucose and 0.8 g/L furfural.** Error bars represent the standard deviation of the mean (n=3). For carbon balance estimation, carbon used for biomass, ethanol and glycerol production were estimated by the molar ratio of carbon in biomass, ethanol and glycerol to carbon in consumed glucose respectively. An elemental formula CH_1.65_O_0.54_N_0.14_ was used to calculate the carbon molar mass in biomass [[2](#_ENREF_2)]. Carbon used for CO_2_ and other byproducts formation was not measured but calculated based on the theoretical assumption.

| Strain | Biomass (g/L) | Residual glucose (g/L) | Ethanol (g/L) | Glycerol  (g/L) | Ethanol productivity [g/(L·h)] | Ethanol yield (g/g) | Carbon balance estimation | | | | | | |
| --- | --- | --- | --- | --- | --- | --- | --- | --- | --- | --- | --- | --- | --- |
|  |  |  |  |  |  |  | Ethanol | Glycerol | | Biomass | | CO_2_ and other byproducts |  |
| BAD | 0.87 ± 0.02 | 14.03 ± 0.03 | 2.53 ± 0.06 | 0.29 ± 0.01 | 0.08 ± 0.00 | 0.13 ± 0.00 | 0.61 ± 0.02 | | 0.05 ± 0.00 | | 0.20 ± 0.00 | 0.15 ± 0.02 |  |
| *siz1Δ* | 3.45 ± 0.05 | 0 ± 0.00 | 9.00 ± 0.30 | 0.73 ± 0.03 | 0.30 ± 0.01 | 0.46 ± 0.02 | 0.60 ± 0.02 | | 0.04 ± 0.00 | | 0.22 ± 0.00 | 0.14 ± 0.02 |  |

# References

1. Shao Z, Zhao H, Zhao H: **DNA assembler, an in vivo genetic method for rapid construction of biochemical pathways.** *Nucleic Acids Res* 2009, **37:**e16.

2. Von Stockar U, Liu JS: **Does microbial life always feed on negative entropy? Thermodynamic analysis of microbial growth.** *Biochim Biophys Acta Bioenerg* 1999, **1412:**191-211.
